# Factors influencing uptake of diabetes health screening: a mixed methods study in Asian population

**DOI:** 10.1186/s12889-022-13914-2

**Published:** 2022-08-09

**Authors:** P. V. AshaRani, Fiona Devi, Peizhi Wang, Edimansyah Abdin, Yunjue Zhang, Kumarasan Roystonn, Anitha Jeyagurunathan, Mythily Subramaniam

**Affiliations:** 1 Research Division, Institute of Mental Health, 10 Buangkok View, Singapore, 539747 Singapore; 2 Saw Swee Hock School of Public Health and Department of Medicine, National University of Singapore, Singapore, 117549 Singapore

**Keywords:** Diabetes, Health screening, Barriers, Facilitators, Mixed method, Qualitative

## Abstract

**Background:**

Health screens are the cornerstones for health promotion and preventive interventions at a community level. This study investigated the barriers and facilitators to the uptake of diabetes health screening in the general population of Singapore.

**Methods:**

In this mixed methods study, participants without diabetes were recruited from the general population. The quantitative phase (*n* = 2459) included face to face survey of participants selected through disproportionate stratified random sampling. Those who participated in the quantitative survey were then randomly chosen for a one-to-one semi-structured interview (*n* = 30).

**Results:**

Among the survey respondents, 73.09% (*n* = 1777) had attended a diabetes health screening in their lifetime whilst 42.36% (*n* = 1090) and 57.64% (*n* = 1328, *p* < 0.0001) attended the health screens regularly (every 12 months) and irregularly, respectively. A significantly higher proportion of older adults (≥ 40 years) attended regular diabetes health screening compared to younger adults (less than 40 years; 55.59% vs 24.90%, *p* < 0.001). The top 3 reasons for attending regular health screens were to detect diabetes early, to make lifestyle changes in case of a diagnosis and being health conscious. Qualitative interviews identified similar issues and complex nuances that influenced the uptake of regular diabetes health screening. Several personal factors (laziness, self-reliance, psychological factors, etc.), competing priorities, fatalistic beliefs, affordability, misconceptions about the screens, and appointment related factors (inconvenient location, time, etc.) were identified as barriers, while affordable screens, sense of personal responsibility, perception of susceptibility /risk, role of healthcare team (e.g. reminders and prescheduled appointments) and personal factors (e.g. age, family, etc.) were facilitators. Age, household income, ethnicity and educational level were associated with the uptake of regular diabetes health screening.

**Conclusion:**

The uptake of regular diabetes health screening can be improved. Several barriers and enablers to the uptake of diabetes health screening were identified which should be addressed by the policy makers to alleviate misconceptions and create greater awareness of the importance of the programme that will improve participation.

**Supplementary Information:**

The online version contains supplementary material available at 10.1186/s12889-022-13914-2.

## Background

Diabetes is a rapidly emerging global health concern with 537 million people estimated to have the disease [1]. The number is expected to increase by 46% in 2045 globally with highest growth estimated for Africa (134%), Middle East & North Africa (87%) and South-East Asia (68%) [2, 3] As the number of cases with diabetes go up, the economic cost due to diabetes is also expected to increase from the current estimate of 966 billion USD in 2021 to 1054 billion by 2045 [3]. While these statistics represent diagnosed cases of diabetes, globally one in two adults aged 20–79 years are undiagnosed and unaware that they have type 2 Diabetes Mellitus (T2DM) (44.7%) [2]. Untreated diabetes can reduce one’s life expectancy and lead to visual impairments, stroke, diseases of kidney and heart, etc. [3]. Nearly 6.8 million people (20–79 years of age) died due to diabetes and its complications in 2021 (excluding mortality associated with COVID-19) and about one third of these deaths happened in people of the working age group [1]. Considering the dire consequences of long term diabetes on mortality, morbidity and global economy, preventive efforts to ramp up early detection to ensure timely and adequate care are essential.

Accumulating evidence confirms that T2DM can be prevented or delayed [4, 5]. The World Health Organisation has explicitly stated the need for a population level prevention to tackle the modifiable risk factors of diabetes (such as, unhealthy diet, sedentary lifestyle, lack of physical activity, smoking), provision of targeted screening for those at high risk of developing T2DM through blood glucose monitoring, implementation of risk assessments and interventions to delay the progress of diabetes [6], and opportunistic screening for high risk individuals who present with symptoms to healthcare [6, 7]. Policy makers and healthcare providers adopt different policies and programmes for diabetes screening depending on the prevalence of the disease, severity of the cases, and the choice of screening tests [8]. In current practice, prescreening assessments such as waist hip ratios and risk assessment calculators are used as indicators of ‘at risk’ individuals worldwide [6]. The diagnosis of diabetes is made through fasting blood glucose levels (≥ 7.0 mmol/L) or 2 h plasma glucose level (≥ 11.1 mmol/L; oral glucose tolerance test) or glycated hemoglobin levels (≥ 6.5%) [6]. These tests are often offered as a basic comprehensive screening package comprising anthropometric measures and monitoring of lipids, glucose and blood pressure.

Creanor et al. [9] showed that among the primary care and dental service settings, only 20% of the respondents had undergone diabetes screening in the past 12 months and 61% were never screened for diabetes. The authors also showed that 82% of those in the primary care setting were willing to undergo diabetes screening if offered to them. Eborall and colleagues [10] investigated the reasons for lower uptake through a qualitative study design and evidenced that perceptions about diabetes candidacy (the thought that it affects older age, has hereditary and lifestyle influences), perception of severity (e.g. diabetes is not as risky as other medical conditions), being busy, longer appointment time at the screening, apprehension about the screening results and the reluctance to take the oral glucose tolerance tests were barriers to the screening. A systematic review of nine studies conducted in England, investigated the barriers to the uptake of National Health Systems (NHS) health screening [11] and identified lack of awareness towards the health screens, lack of understanding of the preventive purpose of the health screens, lack of interest in the programme (e.g. peer influence, afraid of the screening results, not keen, etc.), time constraints and competing priorities, difficulty in getting an appointment for the health screen and concerns about the screening setting (e.g. privacy and confidentiality) as barriers to the uptake. Another systematic review of 39 studies reported similar barriers for cardio metabolic screens [12]. The review identified younger age, lower education, negative attitudes such as not wanting to do checkups, not wanting to know, lack of time, lack of awareness, lack of perceived severity and appointment related issues as barriers while feeling responsible for one's own health and concerns about health status acted as enablers for the health screens.

A systematic review involving 14 articles from Singapore evidenced lower participation in health screening with concerns over cost, misconceptions about the purpose of health screening, lack of trust in the health care system and lack of time of the clinicians, as major barriers [13]. The socioeconomic status was associated with poorer health outcomes and screening attendance [13, 14]. Specifically, attendance to diabetes health screening was 38.8% vs 59.6% in low and high socioeconomic groups, respectively [13]. In view of the higher prevalence of diabetes (8.6% in 2010 and 9.5% in 2020, in those aged 18–74 years), the Ministry of Health, Singapore declared a ‘War on Diabetes (WoD)’ campaign in 2016 to support the ongoing efforts to prevent diabetes [15, 16]. The campaign includes elements such as education and awareness, healthy living (supportive environment, social movements (e.g. drink water campaigns, health cooking programmes) and incentives to support healthy lifestyle), enhancing the skills of caregivers and healthcare team, peer and community support and affordable medical care costs, and improving outcomes (raising health insurances and patient outcome funding models based on findings through programme evaluations). The policy core of WoD is promoting physical activity, healthy dietary habits, and improving early diabetes screening and management [15].

The diabetes prevention programme spearheaded by the Health Promotion Board Singapore [17], a subsidiary of the Ministry of Health targets those above 18 years old through a two-step programme; Diabetes Risk Assessments (DRA, 18–39 years) and subsidised blood glucose tests (under '*Screen For Life'* (SFL)) *screen for life* programme for those who are ≥ 40 years of age). Those who score high on DRA are eligible for the subsidised diabetes screening. The national policy recommends regular screening at least every 3 years [17] or more frequently as recommended by the healthcare provider [18]. Nonetheless, annual screening packages are offered by many workplaces and community partners either free of charge or at a subsidised rate in Singapore for those aged 18 years and older. While recommended guidelines target those who are 40 years and above, the global prevalence of diabetes in the younger age group is on the rise. The prevalence of diabetes in 2021 was 2.2% among those aged 20–24 years [1]. The prevalence increases with age, and approximately 7% of those aged 35–39 years were diagnosed to have diabetes in 2021. Therefore, younger age groups cannot be neglected in terms of anti-diabetes campaigns.

The previous studies on barriers and enablers of diabetes health screens were conducted in specific cohorts (e.g. men, patients attending GP clinics) and not much research has been done to understand context of these finding as many of the studies were quantitative in nature, focusing on health seeking behaviours in general. There is a dearth of literature on the barriers and facilitators specific for regular diabetes health screens. This information is very crucial to compliment the ongoing preventive initiatives that will help the policy makers to make necessary changes to improve the uptake of the programme by addressing these barriers. Given that these barriers could be different in different settings and geographical regions; more studies need to be conducted to understand the user’s perspective about the diabetes screening programmes. This mixed methods study aims to understand the barriers and facilitators to the uptake of diabetes health screening in the general population in Singapore.

## Methods

The study adopted a concurrent triangulation mixed method design to capture the information from the general public who did not have a diagnosis of diabetes (as diagnosed by a clinician). The study included a quantitative (*n* = 2459) and a subsequent qualitative phase (*n* = 30) where both data sets were handled equally without prioritising one over the other. A parallel data analysis approach was used to combine the qualitative and quantitative data where the analysis was converged only during the interpretation stage (distinct data collection and analysis for quantitative and qualitative data). This approach provides enriched data that will give a better understanding of the phenomenon under study [19].

### Quantitative survey

The quantitative phase consisted of a nationwide survey conducted between February 2019-September 2020 [20]. The details of the study methodology are reported elsewhere [20]. The survey captured the knowledge, attitudes and protective practices towards diabetes among the general public. The study recruited a total of 2895 respondents; of which 2459 participants had no diabetes and were included in this study. The survey had a response rate of 66.2%.

Briefly, the participants (citizens or permanent residents) who were aged 18 years or above and speaking either English, Chinese, Malay or Tamil were recruited through a disproportionate stratified random sampling. An invitation letter was sent to all the selected participants before the home visit by the interviewers who captured the data through Computer Assisted Personal Interviews (CAPI). The study related procedures were reviewed and approved by the Domain Specific Review Board of the National Healthcare Group (DSRB; Ref: 2018/00430). Written informed consent was taken from all the participants.

#### Questionnaire for quantitative survey

All the questionnaires used in the study were cognitively tested in the local population [20]. While the recommended screening age is ≥ 40 years under the  SFL subsidised programme, (unless at a higher risk of developing diabetes), [17] those who were 18 years or above were included in the current study as annual health screenings are commonly offered by workplaces and healthcare providers to willing participants. The uptake of diabetes specific health screening was captured through the question “Have you ever attended a health screening for diabetes or a blood sugar test?” The response options included, ‘Yes’, ‘No’ and ‘Don’t know’. For the current analysis, ‘No’ and ‘Don't know’ were combined as one category. Those who answered ‘Yes’ were asked “how frequently do you attend diabetes health screening”? The answer was captured in months, in open text format. Those who attended screening every 12 months or less were classified as having ‘attended regular screening’ and those who reported undergoing a screening more than 12 months apart were considered as having ‘not attended regular screening’. The barriers (10 items) and facilitators (12 items) were further explored through responses to a series of statements that are relevant to the Singapore context, about the uptake of diabetes health screening. Motivators for attending health screening regularly were also captured through similar statements and the response scale ranged from strongly disagree to strongly agree with the option for ‘neutral’ response in the middle. These questionnaires were developed in house through literature review and in consultation with leading diabetologists. The questionnaires were cognitively tested in members of public to adapt it to the local population and to capture any additional items in the response options that are locally relevant.

The quantitative survey was intended to capture the proportion of residents attending regular diabetes health screening and to identify the common barriers and facilitators to attend health screening. Qualitative interviews were conducted to capture additional barriers which are relevant locally, and to understand the context, breadth and depth of the barriers and facilitators captured in the quantitative phase.

### Qualitative interviews

Participants from the quantitative survey, aged 21 years and above, who gave written permission for re-contact for future research, were stratified according to their age (≥ 40 years and < 40 years), gender, and ethnicity (Chinese, Malay, Indians, and others). Participants were then randomly chosen using an online randomisation software [21] and recruited into the qualitative phase. In total 30 one-to-one semi-structured interviews were conducted during the period August 2020 to March 2021. There were 20 interviews in English (*n* = 20) and the rest in local languages (Chinese (*n* = 4), Malay (*n* = 3), and Tamil (*n* = 3)). The interviews lasted for 1 to 1.5 h and were conducted either face to face or through video-conferencing. Written consent was taken from all the participants as per the approved ethics protocol (DSRB ref:2019/00926) and the interviews were audio recorded and then transcribed verbatim by professional transcription firms and cross checked by the study team for quality and accuracy. A multiple triangulation (method and researcher triangulations) was adopted to ensure the credibility of the data. The team members who conducted qualitative interviews were researchers with different cultural background and experience. The interviewers assured the participants of their neutrality although they were affiliated to healthcare institutions as well as the steps taken to ensure confidentiality. While some of them were caregivers or had close contact with a person with diabetes, none of them were providing clinical care to people with diabetes. The familiarity with the healthcare system and certain nuances of diabetes diagnosis and care that were unique to the Singapore system were thus well understood by the interviewers allowing them to establish a good rapport with the interviewees. To the extent possible the ethnicity of the interviewer was matched to that of the respondent, which eased the discussion on cultural barriers and allowed the use of words in the native language where needed to enhance the narratives. Six interviewers conducted the 30 interviews with any two interviewers per session and seven researchers were involved in the checking and interpretation of the data.

### Analysis

#### Quantitative analysis

Descriptive analysis was performed for the quantitative data. Survey weights were employed to adjust for the age and ethnicity to adjust for oversampling and non-response so that the results are representative of the general population. Logistic regression was performed to understand the sociodemographic correlates of the irregular uptake of diabetes health screening. The analysis was performed using with Stata version 15.

#### Qualitative analysis

The qualitative data was analysed using Nvivo V.11 (QSR International. NVivo V.11). An inductive content analysis was performed by breaking down the data into small groups and coding them based on the contents to derive themes [22]. The codes identified through initial screening were discussed and modified. The codebook was constructed after 20 interviews. A total of seven researchers reviewed the transcripts to construct the code book (ZY, AR, FD, WP, KR, AJ and MS). Any disagreements in the definition or inclusion of codes were resolved between the researchers through discussions and literature review. Data saturation was achieved at 20 interviews. Additional 10 interviews in other local languages (Chinese, Malay and Tamil) were conducted to ensure that the perspectives of people who were not native English speakers are taken into account. Thus, a total of 30 interviews covered the wider perspectives of different participants with different socio demographic characteristics (maximum variation sample-equal representation from different gender, age groups, ethnicities and languages) to achieve a richer data that provide a better understanding of the phenomenon [23]. An inter-rater reliability of 0.87 (Cohen's κ coefficient) was achieved between AR, FD and WP as calculated using the NVivo 11. The three interviewers (AR, FD and WP) coded all the transcripts.

## Results

### Quantitative survey

#### Sociodemographic characteristics

The sociodemographic information of the sample is included in the additional file 1: Table S1.

Majority of the respondents were Chinese (76.91%, *n* = 731) and employed (72.44%, *n* = 1781). There was an equal representation across the different age groups and gender.

#### Uptake of diabetes health screen

Among the survey respondents (*N* = 2459), 73.09% (*n* = 1777) stated that they had attended a diabetes health screening in their lifetime (≥ 40 years:85%; < 40 years: 57.28%; *p* value ≤ 0.0001). Nearly half of the population (42.36%, *n* = 1090) attended regular diabetes health screening (every 12 months or less) while 57.64% (*n* = 1328, *p* < 0.0001) did not attend the health screening regularly. A significantly higher proportion of those aged ≥ 40 years attended regular diabetes health screening (55.59% vs 24.9%; *p* < 0.0001) than those aged below 40 years.

**Fig. 1:**
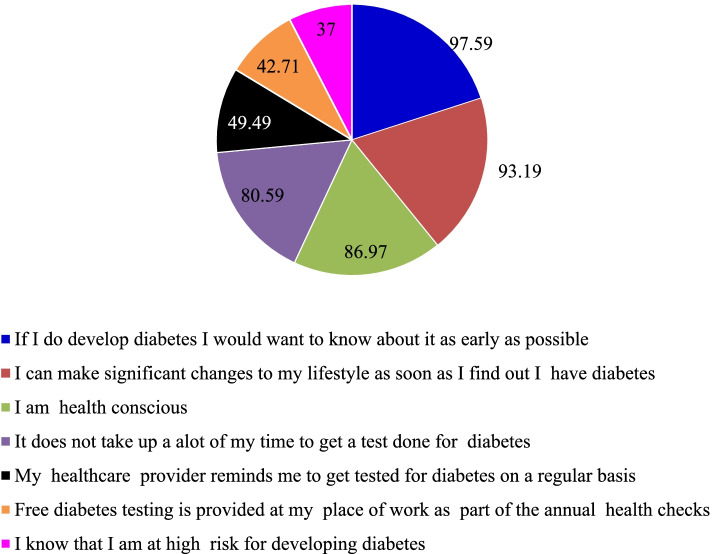
Participants’ endorsed reasons for attending regular diabetes health screening (overall sample)

Among the reasons for attending regular diabetes health screening, the top 3 reasons included a) wish to know early if they develop diabetes (97.59%, *n* = 1069) b) to help to make significant changes to lifestyle if detected early (93.19%, *n* = 1022), and c) they were health conscious (86.97%, *n* = 962). The top 3 reasons identified by different age groups (≥ 40 years and < 40 years) remained the same with no major differences noted between the groups (additional file 1: Table S2). Compared to those below 40 years of age, a significantly higher number of those ≥ 40 years endorsed that they attended diabetes health screening as they were health conscious (90.2% vs 77.5%; *p* = 0.0003) and their healthcare provider reminded them to get tested on a regular basis (56.4% vs 29.2%; *p* = 0.000). Similarly, a significantly higher number of those below 40 years endorsed that they attended regular diabetes health screening as part of the free annual screening is provided at their workplace (60.1% vs 36.6%; *p* = 0.000). The top 3 barriers identified included a) do not know where to get free diabetes health screening (44.85%, *n *= 595), b) habit of procrastination delayed the testing (32.23%, *n* = 413), c) worry that the diagnosis would have a negative impact on their life (23.48%, *n* = 333), d) they will have to make significant changes to their life if they find out they have diabetes (23.48%, *n* = 333). The motivators (top 3 among the list) included a) follow up arrangement by screening centers to consult a healthcare professional (88.73%, *n* = 1180), b) cost of diabetes treatment explained clearly beforehand (86.49%, *n* = 1160), and c) a trained healthcare professional would explain the meaning of the test results (81.83%, *n* = 843). The detailed list of questionnaires and responses for all statements are indicated in the Fig. 1 and Table 1. The barriers and motivators as endorsed by different age groups are largely the same and are included in the additional file 1: table S3.

**Table 1:** Quantitative survey: Participant reported barriers and motivators

*I don’t attend diabetes screening regularly because:*	Weighted %	n	*I would be motivated to attend the Diabetes health screening more regularly if:*	Weighted %	n
I do not know where to get free diabetes testing	44.85	595	On being diagnosed as suffering from diabetes, there is follow up in terms of a polyclinic/ GP/ specialist appointment arranged by the health screening centre	88.73	1180
My habit to put things off has resulted in my delaying the decision to get tested	32.23	413	On being diagnosed as suffering from diabetes, the cost of treatment is clearly explained to me	86.49	1160
If I find out that I have diabetes, I will have to make significant changes in my life	23.48	333	A trained health personnel should clearly explain the meaning of my test results	81.83	843
If I find out that I have diabetes, it would have a negative impact on my life	23.44	319	My GP encouraged me to go for health screening	78.8	1057
It will take a lot of my time to get a diabetes test	17.93	255	The amount of time I would need to get a diabetes test is clearly specified	75.46	1042
Even if I find out that I have diabetes I don’t think I can afford the treatment	16.37	233	I am clearly told where I can access free diabetes testing	68.7	965
The pain from a diabetes test (finger stick or blood draw) makes me reluctant to get tested for diabetes	12.57	161	The health screening tests can be held over weekends	65.19	899
I do not like to fast overnight which is needed for a proper health screening	12.18	149	My family and friends accompanied me for the health screening	53.51	762
I do not want to know if I have diabetes	10.17	151	I am given incentives in the form of supermarket vouchers	51.35	737
Knowing that I am at high risk for developing diabetes makes it difficult for me to get tested	5.935	86	The screening was held in association with workout sessions such as yoga classes or group aerobics which are age appropriate	50.97	714
			I am given incentives in the form of free gym classes	49.01	724
			The screening was held in association with workshops on healthy cooking	41.87	631

#### Factors associated with the uptake of regular diabetes health screening

Age, household income, ethnicity and educational level were associated with the uptake of regular diabetes health screening in the overall sample. Older adults had higher odds of attending regular diabetes health screening compared to those aged 18–34 years (Table 2). (*p* < 0.000). Compared to Chinese, Indians (OR:1.73, 95% CI: 1.34–2.24, *p* < 0.000) and Malays (OR: 1.35, 95% CI: 1.04–1.75, *p* = 0.024) had higher odds of attending regular diabetes health screening. Compared to those with higher education (Degree and above) those with lower education (Secondary school: OR: 0.59, 95% CI:0.37–0.95, *p* = 0.029; Vocational institute/ITE: OR: 0.54, 95% CI:0.31–0.95, *p* = 0.031) had lower odds of attending diabetes health screening regularly. Similarly, compared to those with a salary of S$10, 000 and above, those with no income (OR: 0.22, 95% CI: 0.11–0.48, *p* < 0.000) or lower income (S$2,000–3,999; OR: 0.30, 95% CI: 0.17–0.53, *p* < 0.000) had lower odds of attending regular diabetes health screening.

**Table 2:** Sociodemographic correlates of regular diabetes health screening

Independent variables	Overall				Below 40 years				40 years and above			
	**OR**	***P* value**	**95% CI**		**OR**	***P* value**	**95% CI**		**OR**	**P value**	**95% CI**	
			Lower	Upper			Lower	Upper			Lower	Upper
**Age groups**												
18 to 34 (Ref)												
** 35–49**	**3.24**	**0.000**	**2.23**	**4.71**	**-**	**-**	**-**	**-**	**-**	**-**	**-**	**-**
** 50–64**	**7.01**	**0.000**	**4.54**	**10.80**	**-**	**-**	**-**	**-**	**-**	**-**	**-**	**-**
** 65 and above**	**16.91**	**0.000**	**9.75**	**29.35**	**-**	**-**	**-**	**-**	**-**	**-**	**-**	**-**
**Gender**												
** Female (Ref)**												
Male	0.88	0.374	0.67	1.16	0.90	0.656	0.56	1.44	1.20	0.308	0.85	1.70
**Ethnicity**												
Chinese (Ref)												
** Malay**	**1.35**	**0.024**	**1.04**	**1.76**	1.39	0.147	0.89	2.17	1.16	0.349	0.85	1.58
** Indian**	**1.72**	**0.000**	**1.33**	**2.22**	**2.12**	**0.000**	**1.40**	**3.20**	**1.53**	**0.010**	**1.10**	**2.11**
Others	1.40	0.113	0.92	2.14	1.30	0.473	0.64	2.65	1.55	0.115	0.90	2.67
**Education**												
Degree and above (Ref)												
Primary and below	0.77	0.356	0.44	1.35	0.44	0.190	0.13	1.49	1.60	0.135	0.86	2.98
** Secondary**	**0.57**	**0.020**	**0.35**	**0.91**	**0.36**	**0.025**	**0.15**	**0.88**	1.08	0.798	0.59	1.97
Pre-U/Junior college	0.48	0.073	0.22	1.07	0.59	0.461	0.14	2.41	0.87	0.763	0.34	2.20
Vocational institute/ITE^a^	**0.52**	**0.023**	**0.29**	**0.91**	**0.36**	**0.015**	**0.16**	**0.82**	1.14	0.783	0.45	2.87
Diploma	0.70	0.106	0.45	1.08	0.73	0.317	0.39	1.36	0.86	0.629	0.46	1.60
**Employment**												
Employed (Ref)												
Economically inactive	**1.51**	**0.050**	**1.00**	**2.28**	0.79	0.621	0.31	2.02	**2.55**	**0.000**	**1.61**	**4.03**
Unemployed	1.04	0.923	0.50	2.15	0.91	0.848	0.33	2.48	1.08	0.868	0.42	2.77
**Monthly personal Income (in Singapore dollars)**												
10,000 and above (Ref)												
** No income**	**0.33**	**0.013**	**0.14**	**0.79**	**0.10**	**0.017**	**0.01**	**0.66**	0.40	0.092	0.14	1.16
** Below 2,000**	**0.44**	**0.026**	**0.21**	**0.91**	**0.16**	**0.028**	**0.03**	**0.82**	0.49	0.087	0.22	1.11
2000 to 3,999	0.94	0.855	0.48	1.83	0.33	0.147	0.07	1.48	0.83	0.643	0.38	1.81
4000 to 5,999	0.91	0.774	0.46	1.78	0.46	0.311	0.10	2.06	0.71	0.390	0.32	1.56
6000 to 9999	1.81	0.112	0.87	3.77	0.70	0.656	0.15	3.33	1.79	0.178	0.77	4.20

^a^Institute of Technical Education

Indians had higher odds of attending regular diabetes health screening compared to Chinese in both age groups (Below 40 years: OR: 2.12, 95% CI:1.40–3.20, *p* = 0.000; ≥ 40 years: OR: 1.53, 95% CI:1.10–2.11, *p* = 0.01). Those with lower education status (Secondary school: OR: 0.36, 95% CI:0.15–0.88, *p* = 0.025; Vocational institute/ITE: OR: 0.36, 95% CI:0.16–0.82, *p* = 0.015) had lower odds of attending regular diabetes health screening (compared to those with higher education) in young adults (below 40 years). Similar to the overall sample, older adults (≥ 40 years) who were economically inactive had higher odds of attending regular health diabetes screening (OR: 2.55, 95% CI:1.61–4.03, *p* = 0.000). Monthly income (lower income) was associated with lower odds of uptake of regular diabetes health screening in young adults, but not in older age group (≥ 40 years).

### Qualitative interviews

In total 30 participants were interviewed. There were 16 males and 14 female participants. Sixteen participants were ≥ 40 years of age. Twelve participants were Chinese, 10 were Malay, 6 were Indians and 2 were from other ethnicities. The broad themes were classified into 3 major categories: a) barriers to regular diabetes health screening, b) facilitators/ motivators of regular diabetes health screening and c) suggestions to overcome the barriers to uptake of regular diabetes health screening.

#### Barriers to the uptake of regular diabetes health screening

Several common barriers to attend regular diabetes health screening were reflected in the interviews. The subthemes such as personal factors, fatalistic beliefs, lack of knowledge, misconceptions, affordability, and appointment related factors emerged from the interviews.

##### Personal factors

Attributes of personal factors emerged in nearly 15 interviews which included laziness (*n* = 3), decision to manage their health on their own/self-reliance (*n* = 3), and psychological factors (e.g. attitudes/mindset, lack of motivation/will power, habits, etc.; *n* = 13).

Participants confessed that they were too lazy to go for regular diabetes health screening. They also expressed a reluctance to go to the clinics due to the pandemic and unpleasant healthcare encounters. The healthcare sector was considered to be a high risk place and was generally being avoided unless there was an emergency. The clinician-patient relationship was also a factor in the uptake of regular diabetes health screening. For example, one of the participants who did not attend regular diabetes health screening said:

“First, laziness, and second, I don’t like to see a doctor. So you see, I really seldom see a doctor myself. The ophthalmologist I went to see last year and whatever the doctor told me then, was like nothing needs to be done, and I didn’t need any medicine, we just chatted.” [60 years, Male, Chinese].

Participants’ narratives captured the preference of traditional medicines and practices over western medicines that resulted in limited uptake of regular diabetes health screening. The participant (25 years, Female, Chinese) narrated how his parents relied on Traditional Chinese Medicine (TCM) to monitor and manage their health which became a habit over the years and that they preferred not to seek western medicine for their ailments. Participants (*n* = 4) also felt that they can manage their health on their own (self-reliance) and did not like external factors that forced diabetes health screening. They also felt that having a diagnosis of diabetes would change their life completely which they were not ready for. Moreover, the diagnosis was perceived to bring the attention of the friends and family members to them who would interfere in their life and try to control their lifestyle. Others taking decisions on their behalf and treating them as ‘invalid’, caused unnecessary burden and negative impact on their body and mind. They felt that the changes that follow the diagnosis would add stress to their otherwise normal life. They were worried about losing control of their life and the resistance to external interference made them more reluctant to attend regular diabetes health screening. One participant gave context to this by stating:

"I don't need a doctor to tell me that I may have cholesterol." In some sense, I already may have some feeling based on my diet, my health itself, so I don't want the doctor to actually certify and then say, "Okay, you better start taking this."That's why I say, with the knowledge that we have and the information that's around us, we are able to make the choice and say actually, how to deal with it. “Oh, eat this kind of vegetable more. Drink this kind of thing more," As opposed to having all this regulated by medicine.” [54 years, Male, Indian].

Other personal factors identified through the interview included psychological factors such as individual attitude/mindsets, as narrated by a participant who did not attend regular diabetes health screening as he felt that he had lived his life without any medical conditions until now. He explained that the hassles of attending the health screening led to this mindset. Participants also felt that nothing can be done even if the test shows a specific condition. They gave examples of a diagnosis of a terminal illness like cancer, citing the examples of people who suffered from the illness. Participants felt that such a diagnosis would be devastating and nothing much can be done other than giving up the fight. Such mentality acted as a barrier to the uptake of regular diabetes health screening. Similar narratives were given by other participants who expressed that people do not really worry that much about their health that they do not consider attending regular diabetes health screening. A 54-years old, female (Chinese) participant who attended regular diabetes health screening explained that people’s mentality affects their uptake of blood tests. She explained that:

“I come across a minority who still don't like to go for checkup, my brother-in-law is one of them already. To them it is that, the more you check, the more problem you will diagnose, so they don't like to go for checkup.” (54 years, Female, Chinese).

Participants expressed the view that it was pointless to undergo diabetes health screening as there was nothing much one could do if they were diagnosed with a medical condition. The reasons for this attitude were tendency to easily give up due to lack of will power, reluctance to make changes in their lifestyle, the fear of loss of control over their life, unwillingness to accept that they were sick and to take any medications upon diagnosis. It points towards the reluctance to change and unwillingness to disrupt the status quo.

##### Fatalistic beliefs

This was a frequent subtheme that was identified in nearly half of the interviews (*n* = 12). Participants expressed fears about the outcome of the health screening and that it might reveal life threatening diseases such as cancer. They tried to avoid such potential diagnosis by not attending the health screening. A female interviewee who attended yearly diabetes health screening felt that there is a general reluctance to attend the tests among public because they are afraid of death and are worried that the tests might reveal something serious. The reason behind this fear was fear of death, if diseases such as cancer were diagnosed during the health screening and the cost of treatment for such conditions.

“..it is because when you go for all this checkup they will tell you what are all the sickness that you have which we don’t know for ourselves sometimes when we are sick. Better not to go, so we don’t have to find out and we can continue with our normal lives.” [61 years, Female, Indian].

A participant who attended regular diabetes health screenings shared her recent experience. She explained that she attended the diabetes health screening as a part of her regular healthcare for an existing medical condition, but was otherwise reluctant to go for any health screening. She explained her decision by sharing her fearful experience of a health screening where hematuria was detected in the test which led to further tests, scans, and suggestions for surgery to a point that she refused further interventions. Such fearful experiences added on to her reluctance to take up regular diabetes health screening.

“So I am scared. I don't want to go. If I look normal, I'm okay, okay. Like now, the urine test, doctor says, oh, my urine has protein, have blood you know, but must use a microscope. That's why they ask, "Can you see your urine’s color?" I said it is very clear. "Not orange?" "No. Not orange. Very clear, Very nice color." "No bubble?" "No bubble." But how come? So they wanted to find out. So they asked me to go for an ultrasound. And then they want to see whether both my kidneys are the same size. Okay. They are the same size, nothing. They still were not satisfied because they still found blood and protein in my urine. So they wanted to do a minor operation behind through my kidney. They wanted to check whether there's leaking or whatever.” [55 years, Female, Malay].

##### Misconceptions and lack of knowledge about regular diabetes health screening

This perspective was reflected when participants (*n* = 11) shared their belief that as long as they were healthy and fit, they did not need regular diabetes health screening.

“No. I don’t. Because I feel fit and healthy so why? I only need to go when I am sick…. if we are well we don’t need to go to the doctor. We won’t think of going to see a doctor when we are well. Only if we feel like we have body pain or some head ache then we will go see the doctor, and if they say must check on more things, then you will find out if you have anything. [37 years, Male, Indian].

There was a general misunderstanding that one needs to attend health screening only when they felt sick. This belief stemmed from the misconception that they would stay healthy as long as they ate home cooked healthy food and took care of themselves. People were unaware of the prophylactic role of the health screening. Interviewees though that if they were healthy and had no family history of chronic diseases, their genetics would balance the risk of the disease. Younger participants had a misunderstanding that chronic conditions occurred only in older people and thus, they do not have to undergo regular diabetes health screening regularly. All these misunderstandings and lack of knowledge about the prophylactic roles of health screening prevented uptake. A clear knowledge on the purpose of health screening was lacking.

“Most of it is the lack of awareness and the seriousness of doing such check-ups. But, as I mentioned earlier, as long as they are able to walk, see, they feel that's healthy.” [52 years, Male, Indian].

##### Affordability

This subtheme emerged in many interviews (*n* = 10) where participants highlighted the affordability of the health screening as a barrier. They felt that not all insurances cover the cost of health screening and the free health screening conducted by the workplace was not open to all employees (e.g. staffs on contract were not eligible). As people lacked a clear understanding of the cost associated with the screen they were reluctant to go for the screening. Additionally, the socioeconomic status of people was indicated to be a barrier in the uptake of regular diabetes health screening.

An interviewee expressed that he did not attend regular diabetes health screening although he wanted to because of the cost.

“Whenever I see health screening at clinics, then when I saw the price, some of them is– if I want to do a health screening, I will prefer a full body or full health screening, but it won't be cheap, so I will be hesitant to go for it.” [38 years, Male, Chinese].

The concerns regarding affordability extended beyond the health screening to the treatment of health conditions that the screening might reveal. The cost associated with the treatment of the medical conditions were worrying. The job situations amidst the pandemic situations added on to the worry. Participants felt that the medical cost for treatment is high in Singapore and considering the job situation, income status and coverage of insurance, there was a general reluctance to attend the regular diabetes health screening to avoid these hassles. One participant shared the experience of a colleague:

“..And he refuse to do it because. that’s what he fears that they will pick up something and then he will have to go for further investigations, and he will have to pay more. That’s what they worry as well, because financially he will still pretty.. you know heavily burdened by the kids and stuffs like that.” [53 years, Female, Chinese].

##### Appointment related barriers

The attributes for this barrier (*n* = 9) included long waiting time in the clinics, relationship and interaction with the healthcare team, distance to the clinic/need to travel, not knowing how/where to do the diabetes health screening and issues with the confidentiality of the results. Participants expressed that primary care clinics were too crowded and had a long waiting time for the health screening, which was demotivating.

“..but when I saw the queue, the long queue for people attending screening, that actually kills my motivation. Then I just thought that I will forget about it for those people who need it more, they e can go first. [38 years, Male, Chinese].

Unpleasant experiences with the healthcare staffs where staffs who were inexperienced in handling elderly or were unfriendly towards the patients deterred attendance to health screening. Additional issues included the lack of a proper system to remind people about yearly appointments and the absence of proper instructions on how/where to book an appointment. Operating hours of the clinic (clinics were closed on weekends and public holidays) created inconvenience for individuals who were employed as their busy work schedule deterred them from taking time off from work to attend the health screening. Additionally, the accessibility of the clinic was also noted as a barrier, especially for elderly participants who had no one to help them to go the clinic.

##### Competing priorities

Participants also highlighted other competing priorities (*n* = 6) citing the work culture in Singapore which is too intense so that they had to prioritise short term goals over long term goals (health). References were made to the work environment that kept them busy that they chose to work even when they were sick. Work related commitments and the need to take leave from work to attend health screening were barriers to health screening.

“Very, very hard to go because– very hard, even though I am on medical leave I will stay at work”. [34 years, Male, Malay].

#### Motivators/enablers of regular diabetes health screenings

Attributes of motivators emerged across six areas which included sense of personal responsibility, affordable health screens, perceived risk factor, role of healthcare team, and awareness.

##### Sense of personal responsibility

It was apparent that the respondents (*n* = 23) realised the need to monitor themselves to ensure that they were healthy. There was a general sense of responsibility towards self and their family to stay healthy. The reasons included having young children who were dependent on them and fear of the consequences of an undetected condition. There was also an inherent interest in tracking how their health was progressing over the years through the health checks. Such regular monitoring was perceived to be necessary for taking timely action. Hence, regular health screens acted as a reassurance that everything was in order and they were healthy.

“..I think it is also good to monitor because body can change any time. So if do have a yearly screening, at least, you have a baseline data to see if there's some increase and all those things, you can get treatment early.” [54 years, Female, Chinese,].

Another motivator for attending regular diabetes health screening was the perception of the severity of chronic condition where people realised that certain diseases if detected late could be life threatening and thus one should detect them early for effective treatment. This was sometimes triggered by stressful events and symptoms of minor ailments. Witnessing adverse experiences of those in their social circle also encouraged uptake. This worry was expressed as.

“I have people around me like having all these kinds of things pop out. So, I think most importantly, is that you have to monitor yourself. Do a regular checkup and when you are not well, you have to go and see a doc yourself.” [53 years, Female, Chinese].

##### Affordable diabetes health screenings

The majority of the participants (n = 17) emphasised the importance of affordable health screens as a motivator. Participants highlighted that having subsidised or free health screens would motivate them to attend regular screening. Participants who had attended regular diabetes health screening did so as their insurance covered the cost. Participants also highlighted that their workplace offered free health screen yearly which was a motivator. Additionally, co-payment schemes where the employer paid a certain percentage of the cost incurred for the tests encouraged the uptake.

“..a large part of the reason why I do physical examination is because the company has subsidies” [39 years, Male, Chinese].

The government/insurance subsidies were perceived to be effective in encouraging older generation and those with financial difficulties to go for such screens. Overall, the participants expressed that free blood tests were a strong motivator for attending regular diabetes health screens.

“…if you want to encourage people to go, it must be free then people will be motivated to go.” [53, Female, Indian].

##### Perception of susceptibility/risk

Those who perceived the risks associated with diabetes (susceptibility, risk of complications, health scare, etc.) were motivated to attend regular diabetes health screening (*n* = 12). A family history of diabetes or cancer motivated adoption of a proactive lifestyle with regular follow up health screening. An underlying knowledge about the risk factors was evident. Additionally, pre-existing medical conditions were also emphasised as a motivator. It was evident that having a medical condition created more awareness and acceptance towards screening. Those with existing conditions visited healthcare providers regularly and the frequent communication and follow up created greater awareness. The health screenings were done as a part of these follow-ups which avoided the need for arranging appoints on their own.

“But I think it could also be because of the family medical history. Yeah, they have a very strong history in diabetes and also cancer.” [31 years, Female, Malay].

##### Role of healthcare team

The healthcare setting played a key role in the uptake of regular diabetes health screening (*n* = 11). A steady and open communication and interaction with the healthcare team was emphasised as a motivator for attending health screening. Through the communication, the questions and misconceptions about health screening could be alleviated. Participants felt that clinicians had a major role in the process where they could assess the risk of the patient and suggest health screening that are suitable.

“..with the consistent reminder and advice, "Oh. You're aging already. You need to do some checkup. It's good for you—all these thinsg." Maybe some people would take up because they will think, "Oh, yeah. I never do for 5 years," or, "I never did for 10 years." Yeah. Maybe it's time to do a check and see.” [54 years, Female, Chinese,]

Friendly healthcare staff who gave information on the available tests and costs were also encouraging. People valued such interactions as it gave them a pleasant healthcare experience. Participants highlighted the need for reminders as they tend to forget when the health screening was due. Prescheduled appointments were useful as it gets integrated with their normal lifestyle and became a routine. Participants also appreciated and recognised the role of the healthcare professionals in creating awareness, scheduling screening, and improving engagement by sending reminders through calls, letters or messages.

“..the polyclinic will text me. So I, the message will tell me when to come.” [71 years, Male, Malay].

##### Personal factors

Nearly 11 interviews reflected this subtheme. Age, awareness, role of family/friends, attitudes and health status were described as personal factors that would motivate them to attend regular diabetes health screening. Participants perceived that diabetes screening was inevitable as they aged. This stemmed from the perception that diabetes affects older people. Having reached middle-old age, they felt that health screening was now inevitable. These emerging situations made people more health conscious.

“..Maybe it’s age. I think it’s almost ready, because I am already in my 60 s. So that’s why I said I was ready.” [60 years, Male, Chinese].

Participants also described that awareness/knowledge created through education motivated them to attend regular diabetes health screening. The role of family was also highlighted by the interviewees. Encouragement and influence of family members not only improved their knowledge but also their uptake of health screening. Family members care about each other and track the health status of each other to give timely advise for help seeking which was identified as an important step.

“I guess if your family is more involved with the process, I think it could encourage– not encourage but it could push more people to have regular follow-ups. But if you're able to bring a family member with you for a screening, then maybe the family member could encourage you to do more follow-ups. Or if you're not very sure of what your condition is, then maybe the family member can help you process the information and help you to make better decisions.” [22 years, Male, Chinese].

#### Suggestions to overcome the barriers to the uptake of diabetes health screenings

Participants gave suggestions to improve the uptake of regular diabetes health screening in Singapore (*n* = 18). The active involvement of the government was suggested to be the most effective way to improve the uptake. While the existing programmes are subsidised, they do not target low SES groups who might find the subsidised schemes a drain on their savings. Thus, interviewees expressed that government could further subsidise the programme and arrange more screening programmes free of charge or at a heavily subsidised rate, held at convenient locations, such as near the housing estates, or at a walking distance from them. Health screening programmes held at accessible locations saved time and helped avoid long waiting times.

While the interviewees were aware of the health screening programmes they felt that it had not reached a wider set of general public, especially the older generation or those with language barriers. Hence, suggestions were made by the participants to publicise it through media or talks held at community level to address the concerns and queries of people, and to educate them more about the need for regular diabetes health screening. Such awareness programmes were perceived to be very effective. Participants also felt that elderly in the community would attend the programmes if help is rendered to them through community level volunteers who could assist them in health screening. Overall, the participants felt that government can play a key role in improving the uptake through engaging community centers and media to publicise the screening and to create an awareness. One participant expressed that.

“Tell them, "Okay, go for–" you can see a lot of, now, mobile apps for senior. They are giving freebies, and last time, they used to promote the CHAS card in the TV. Now, for seniors, they said, "Okay, you go. its free after you are 65." These all in all languages, let them know, and the government in the CCs, they got a lot of ambassadors all to go around, knock the seniors' door. These are all good. We must tell them, "Don't be afraid, the government will bear the cost," things like that.” [58 years, Male, Indian].

Regular screening held at work place was also deemed to be an essential step in improving the uptake. Many of the workplaces currently offer health screening as a part of hiring process, extending this to a more regular yearly programme was perceived to be beneficial especially since people can attend the screening during office hours without the need to take leave. Additional suggestions included granting free leave to attend health screening, in the context that the need to apply leave for health screening discourages people.

Awareness creation was identified as another crucial step which was suggested to be through informal authority other than government as people would show some resistance to authority. Family members, clinicians, schools or media can play their part to raise the awareness towards diabetes and influence them to attend regular diabetes health screening. In schools, teachers can play an important role to create awareness and acceptance starting from a younger age. Participants expressed that the healthcare team can act as a source of information/awareness by explaining the importance of these tests and consequences of the disease. The messages from these sources should also portray the severity of the situation as expressed by an interviewee:

“..So I think that the knowledge when you give it to them, it shouldn't be like too strawberry [laughter]. If you want, you have to really tell them the reality of the situation, "Hey, if you don't get checked, this will happen." Then they will be like, "Oh, shit. Okay. Then I've got to–" Singaporeans are like that [laughter]. Singaporeans are like that. If you don't tell them the reality of the situation, they won't accept it. They'll be in denial. Yeah. You've got to tell them like, "Okay. If you don't get yourself checked, in 5 to 10 years’ time, you might die [laughter]," or I don't know [laughter].” [25 years, Male, Malay].

Celebrity endorsements were also thought to be an effective way to convey the message across to public as they can elicit a positive connotation and will be accepted as a credible programme. Healthcare sector was also perceived to have a major role apart from creating awareness, where they can help in scheduling appointments and doing follow-up calls. Taking a more holistic approach towards healthcare was also thought to change people’s attendance which was expressed as.

“..I think it is nice for healthcare to be marketed as some sort of like- to be more holistic and to be more… what’s the word… like to have them think of it as like something relaxing to do. Like equate a visit to a doctor to like a visit to a spa. Maybe that will make them feel more inclined and more motivated to go.” [25 years, Female, Chinese].

## Discussion

This study examined the barriers and enablers to the uptake of regular diabetes health screening in the general population and found a low uptake with only 42.36% of the population without diabetes (55.59% of those ≥ 40 years and 24.9% of those below 40 years; *p* < 0.0001) attending regular diabetes health screening (every 12 months). This is in agreement with previous population studies from Singapore where only 63.4% attended recommended diabetes health screening (every 3 years), [24]. The reasons for this low uptake included the lower socioeconomic status of the individuals, and lower attendance to primary care clinics (regular follow ups, use of subsidised care, etc.) [13]. A cross sectional population level study in Germany showed higher attendance to health screening among those from high SES [25]. Wong et al. [24] reanalysed the data from the National Health Survey 2010 (Singapore) and identified specific socio demographics groups who were likely to attend regular diabetes health screenings. Those with higher income, higher educational level and belonging to Indian ethnicity had a higher likelihood of attending regular diabetes health screening. Participants in the qualitative segment of the current study endorsed socioeconomic status as a barrier due to the concerns on the cost. The lower rate of uptake among those who were ≥ 40 years (current study) when compared to Wong et al. [24] could be attributed to the definition of regular health screening adopted in the study. We had defined regular health screening as annual health screening, contrary to the 3 year interval adopted by Wong et al. [24].This is because annual diabetes health screening is offered by GPs and workplaces despite the diabetes screening (every 3 years) offered under the national subsidised scheme. Given that the participants were aware of the health screening initiatives, as evident through the interviews; additional steps must be taken to improve the access and encourage participation of all socio demographic groups.

The quantitative survey identified the reasons for the uptake of regular diabetes health screening as being health conscious, wishing to know early if they develop diabetes and to make necessary changes in their life. Similar themes were reported in the current and other qualitative studies where participants who attended regular diabetes health screening assumed a proactive role in health promotion and extended the health promotion beyond screening to the treatment of any conditions that emerge [26]. The qualitative interviews identified a sense of personal responsibility among the participants which led to the uptake of regular diabetes health screening.

The current study identified several personal factors, fatalistic beliefs, misconceptions about the purpose of the health screening, affordability competing priorities, and appointment related barriers to regular diabetes health screening. The results from the quantitative surveys and qualitative interviews complimented each other. Another mixed method study conducted locally among those with low SES reported similar barriers [13]. The study showed waiting time at the clinics, relationship with the health care team, fatalistic beliefs, knowledge, misconception (not knowing the prophylactic role), and work priorities as barriers, which are similar to what was found in the current sample. Participants had several misconceptions about health screening that were rooted in their lack of awareness regarding the prophylactic role. They thought that one needed to attend health screening only if they were sick. This is in agreement with previous reports [14, 27, 28]. There was a general belief among people that ‘as long as you are healthy you do not need to attend any screening’ instead of using the regular diabetes health screening to monitor the health status. This belief is considered a part of the Asian culture [28, 29]. This attitude could reduce the uptake and challenge the efficacy of health promotion initiatives and should be overcome through education and awareness programmes. Wee et al. [30] have shown an increased uptake of cardiovascular health screening following educational interventions in a similar population. These awareness/ educational initiatives should also focus on young adults (below 40 years) as the prevalence of diabetes is on the rise in this age group. One in eight new cases of diabetes are diagnosed in this age group [31]. In Singapore the crude prevalence of type 2 diabetes among those aged 30–39 is 3% [16]. Thus, awareness must be created in young adults that will prepare them to identify early signs of diabetes and to go for regular diabetes health screening, especially when they are at risk.

Cost was a major consideration while considering regular diabetes health screening which was noted in both quantitative and qualitative data. While the screening programmes are available at a subsidised rate through government initiatives such as SFL which is a national level screening programme where citizens need to pay S$0-S$5 per screening visits to participating GP clinics [24]. However, not all individuals are eligible for this subsidy. For example, not all primary care clinics are under the Community Health Assist Scheme (CHAS) that offers SFL. Hence, those who prefer to consult or are already seeking treatment under other private hospitals find it difficult to access this schemes, as indicated by the participants in the qualitative interview. Additionally, the eligibility for SFL programme is based on age. Younger individuals, below 40 years who are proactive and interested in monitoring their health on a regular basis, however, are not eligible for the subsidised programme under SFL unless they are at risk of developing diabetes. Also, the subsidies vary according to the residential status (citizens’ vs permanent residents). Younger individuals, especially those from the low SES group who are at the early stages of their career will have limited savings and thus will prioritise other commitments over regular diabetes health screening. Similarly, annual health screening conducted by the workplace is not open to all employees. The cost considerations in older adults, despite the available subsidised programs could be due to the lack of knowledge about such programmes. Furthermore, the concern over cost extends not only to cost of screening, but also includes cost for treatment if the health screening picks up any chronic conditions. Therefore, subsidised screening programms alone might not be effective in improving the uptake of regular diabetes health screening. Hoebel et al. [25] showed that having sufficient insurance coverage could improve the uptake of regular diabetes health screening. Malhotra et al., [32], in their mixed method study conducted in Singapore, on the barriers to uptake of cancer screening identified cost as a main issue despite the subsidised health screening programmes. The participants expressed that offering the health screening free of charge, letting polyclinics to handle the appointments, using an insurance to cover the entire cost, and lowering the insurance premium for those who attend health screening would motivate them to attend regular cancer screening. Revision of the existing insurance coverage to accommodate the health screening costs while providing enhanced protection at a lower premium could ease the fear of financial burden among the population.

Appointment related factors such as attitudes of healthcare professionals, inconvenient timing, distance, not knowing how to fix the appointment, etc. identified in this study were reported in other local studies exploring attendance to cancer screening programmes [13]. Participants valued patient-care provider relationships and unpleasant experiences such as negative attitude, lack of empathy, and language barriers deterred attendance [33, 34]. Holland et al. [35] studied the preventive health attendance of men and showed that personalised reminder systems targeting the patient and family members improved the uptake of the health screening. This is in agreement with our results where participants emphasised that reminders and follow up by the healthcare team could improve participation in the regular diabetes health screening. The quantitative data from the current study is in agreement with the reports with strong emphasis on the role of clinicians in explaining the details of the tests such as cost, test results, follow ups, and acting as source of motivation. Clinicians can influence the participation of the patients in health screening. Rushed consultation and lack of clinician-patient rapport discourages participation [14]. Given the long standing relationship of patients with the primary care team who are the first line of healthcare contact, the clinicians who have up to date information on the health status of the patients should build rapport with the them during consultations, spend time to discuss and recommend health screening to the patient to improve their attendance [12]. Difficulties with arranging appointments among employed individuals was another barrier identified in the study due to the busy schedule, need to travel and clinic closure on public holidays/weekends. This was also highlighted in other studies [36]. While many of the workplaces offer annual medical checkups, not all employees are eligible or have other engagements that prevent them from attending the health screening on scheduled days. Thus changes should be made to promote attendance at workplace screening or at the primary care. This can be done by offering the screening to all the employees and those who wish to attend health screening at the primary care centres should be given time-off and subsidy (regardless of the age) to improve the attendance.

A systematic review of 53 qualitative, 44 quantitative and 6 mixed methods studies identified several barriers and facilitators of health screening [34]. The review showed that fatalistic beliefs, negative attitudes (laziness, procrastination) and avoiding or denying diseases were barriers to the uptake of health screenings. Our quantitative and qualitative results are in agreement with these reports and emphasise the need for educational initiatives and awareness programme to overcome the fear. Several suggestions were put forward by the participants which included onsite health screening programms held at the housing estates or other convenient locations, advertisement, and celebrity endorsement of the programmes to enhance the reach which should be considered while designing community level interventions.

We have also observed that younger adults, those of Chinese ethnicity compared to those belonging to Indian and Malay ethnicity, and those with lower education and income are less likely to attend regular diabetes health screening (overall sample). This is in line with previous studies that have shown similar results [12, 24]. We have also noted that compared to younger adults (18–34 years), those who are ≥ 35 years, had higher likelihood of attending regular diabetes screen, the odds increasing with age (35–49 < 50–64 < 65 years; overall sample, Table 2). The uptake of diabetes health screening was significantly lower in the young age group (18–39 years). This could be due to the lower perception of the need for diabetes screening among younger adults as endorsed by the participants in the qualitative segment. Although all age groups are exposed to the anti-diabetes campaigns, the subsidised programmes and educational initiatives are targeted at those who are above 40 years which could be another reason for the lower uptake in this age group. Additionally, young adults (below 40 years) with lower education and income had lower odds of attending regular diabetes health screening whereas, older adults (≥ 40 years) who were economically inactive had higher odds of regular uptake. The older adults are eligible for government subsidies for health screening and various other assistance schemes based on their economic status which could be the reason behind the higher uptake. These sociodemographic groups who are unlikely to attend regular screening should be targeted for health promotion programmes to improve their awareness and uptake of regular diabetes health screening. Those with lower education and income might have misconceptions and concerns about the cost of the screening as expressed in the qualitative interviews, which should be addressed in the outreach programmes, in a language comprehensible to them. Given that the prevalence of diabetes is rising globally among all age groups, future research should also focus more on younger adults to understand the perceived risk and severity of diabetes in this age group so as to design effective interventions to achieve a better prevention of diabetes across the globe.

This study has several strengths including the disproportionate stratified random sampling which improves the generalisability of the results. Nonetheless, the cross sectional nature of the quantitative segment is a limitation as people's health behaviour changes with time and cross sectional studies do not capture these changing trends. The qualitative study selected individuals randomly using a random number generator which is an additional strength. Additionally, the study was conducted in different local languages to capture a wider view of the population, thus overcoming language barriers. The cross sectional nature of the study limits deducing any causal relationships.

## Conclusion

A clearer and targeted communication of the health screening programmes is warranted for both age groups which should include information on how to book an appointment, the time taken for the test, and the cost. Attendance to the programme can be improved through affordable health screenings, educational initiatives to improve the perception of susceptibility/risk, better relationship with the healthcare team and onsite programmes at convenient locations. Several barriers were identified by the participants which were similar in both age groups. These barriers included personal factors, fatalistic beliefs, lack of knowledge, misconceptions regarding health screenings, affordability and other appointment related factors which should be addressed in order to improve the acceptance rates. Appropriate policies should be put in place to ease the concerns on the financial burden for those who receive a diagnosis of a chronic condition during routine health screenings.

## Supplementary Information

**Additional file 1: Table S1.** Sociodemographic characteristics of the sample. **Table S2.** Reasons for attending regular health screening as endorsed by different agegroups. **Table S3.** Participants’ endorsed barriers and enablers for attending regular diabetes health screening according to age groups.

## Data Availability

All the datasets analysed during the study are included in this published article and its supporting information.
