# Supplementary material for: Factors influencing uptake of diabetes health screening: a mixed methods study in Asian population
Source: BMC Public Health. 2022 Aug 9;22:1511. doi: 10.1186/s12889-022-13914-2 (PMC9360713; doi:10.1186/s12889-022-13914-2)
Supplement: Supplementary file 1 — Additional file 1: Table S1. Sociodemographic characteristics of the sample. Table S2. Reasons for attending regular health screening as endorsed by different agegroups. Table S3. Participants’ endorsed barriers and enablers for attending regular diabetes health screening according to age groups. [file 12889_2022_13914_MOESM1_ESM.docx]

**Table 1:** Sociodemographic characteristics of the sample

|  | Overall |  | Below 40 years | | ≥40 years | |
| --- | --- | --- | --- | --- | --- | --- |
|  | Weighted % | n | Weighted % | n | Weighted % | n |
| **Age** |  |  |  |  |  |  |
| 18 to 34 | 32.71 | 817 | - | - | - | - |
| 35 to 49 | 29.82 | 670 | - | - | - | - |
| 50 to 64 | 24.51 | 591 | - | - | - | - |
| 65 and above | 12.95 | 381 | - | - | - | - |
| **Gender** |  |  |  |  |  |  |
| Female | 52.23 | 1258 | 52.68 | 551 | 51.88 | 707 |
| Male | 47.77 | 1201 | 47.32 | 502 | 48.12 | 699 |
| **Ethnicity** |  |  |  |  |  |  |
| Chinese | 76.91 | 731 | 72.59 | 290 | 80.16 | 441 |
| Malay | 12.14 | 811 | 15.28 | 365 | 9.78 | 446 |
| Indian | 7.94 | 725 | 9.35 | 330 | 6.88 | 395 |
| Others | 3.01 | 192 | 2.79 | 68 | 3.18 | 124 |
| **Education** |  |  |  |  |  |  |
| Primary | 18.32 | 456 | 1.94 | 35 | 30.63 | 421 |
| Secondary | 19.65 | 552 | 14.85 | 193 | 23.25 | 359 |
| Pre-U/Junior college | 5.03 | 112 | 6.10 | 59 | 4.22 | 53 |
| Vocation | 6.77 | 241 | 10.61 | 156 | 3.88 | 85 |
| Diploma | 19.03 | 442 | 27.25 | 279 | 12.86 | 163 |
| Degree, professional qualifications and above | 31.21 | 656 | 39.25 | 331 | 25.16 | 325 |
| **Employment** |  |  |  |  |  |  |
| Employed | 72.44 | 1731 | 74.75 | 762 | 70.70 | 969 |
| Economic | 23.72 | 617 | 20.47 | 235 | 26.17 | 382 |
| Unemployed | 3.838 | 111 | 4.78 | 56 | 3.13 | 55 |
| **Monthly Income (SGD*)** |  |  |  |  |  |  |
| Below 2,000 | 38.58 | 982 | 32.44 | 371 | 43.18 | 611 |
| 2,000 to 3999 | 25.82 | 627 | 32.13 | 339 | 21.09 | 288 |
| 4,000 to 5999 | 13.88 | 295 | 16.47 | 145 | 11.94 | 150 |
| 6,000 and 9999 | 8.61 | 167 | 8.43 | 61 | 8.74 | 106 |
| 10,000 and above | 6.21 | 104 | 2.09 | 11 | 9.30 | 93 |
| No income | 6.9 | 173 | 8.44 | 86 | 5.75 | 87 |

SGD: Singapore Dollars; * Income category as per previous national surveys [1]

1. Subramaniam M, Abdin E, Vaingankar JA, Shafie S, Chua BY, Sambasivam R, Zhang YJ, Shahwan S, Chang S, Chua HC, Verma S, James L, Kwok KW, Heng D, Chong SA. Tracking the mental health of a nation: prevalence and correlates of mental disorders in the second Singapore mental health study. Epidemiol Psychiatr Sci. 2019 Apr 5;29:e29. doi: 10.1017/S2045796019000179.

Table 2: Reasons for attending regular health screening as endorsed by different age groups.

|  | Below 40 years | | ≥40 years | |  |
| --- | --- | --- | --- | --- | --- |
| ***I attend regular diabetes health screening because..*** | n | Weighted % | n | Weighted % | p value* |
| If I do develop diabetes, I would want to know about it as early as possible | 1069 | 97.7 | 1069 | 97.6 | 0.9420 |
| I can make significant changes to my lifestyle as soon as I find out I have diabetes | 1022 | 92.0 | 1022 | 93.6 | 0.5338 |
| I am health conscious | 962 | 77.5 | 962 | 90.2 | **0.0003** |
| It does not take up a lot of my time to get a test done for diabetes | 892 | 76.4 | 892 | 82.0 | 0.1759 |
| My healthcare provider reminds me to get tested for diabetes on a regular basis | 607 | 29.2 | 607 | 56.4 | **0.0000** |
| Free diabetes testing is provided at my place of work as part of the annual health check up | 498 | 60.1 | 498 | 36.6 | **0.0000** |
| I know that I am at high risk for developing diabetes | 442 | 30.1 | 442 | 39.3 | 0.0501 |

*p value less than 0.05 is considered significant (Chi-Square Test)

Table 3: Participants’ endorsed barriers and enablers for attending regular diabetes health screening according to age groups

|  | Below 40 years | | ≥40 years | |  |  | Below 40 years | | ≥40 years | |  |
| --- | --- | --- | --- | --- | --- | --- | --- | --- | --- | --- | --- |
| ***I don’t attend diabetes screening regularly because..*** | n | Weighted % | n | Weighted % | p value | ***I would be motivated to attend the Diabetes health screening more regularly if…*** | n | Weighted % | n | Weighted % | p value* |
| I do not know where to get free diabetes testing | 360 | 46.6 | 235 | 42.4 | 0.296 | On being diagnosed as suffering from diabetes, there is follow up in terms of a polyclinic/ GP/ specialist appointment arranged by the health screening center | 681 | 89.7 | 499 | 86.7 | 0.223 |
| My habit to put things off has resulted in my delaying the decision to get teste | 237 | 31.5 | 176 | 33.2 | 0.642 | On being diagnosed as suffering from diabetes, the cost of diabetes treatment | 677 | 87.6 | 483 | 84.7 | 0.278 |
| If I find out that I have diabetes, I will have to make significant changes in my life | 155 | 17.3 | 178 | 31.3 | **0.000** | A trained health personnel should clearly explain the meaning of my test results | 494 | 63.4 | 349 | 60.4 | 0.435 |
| If I find out that I have diabetes, it would have a negative impact on my life | 175 | 23.0 | 144 | 24.1 | 0.739 | My GP encouraged me to go for health screening | 587 | 77.3 | 470 | 80.1 | 0.386 |
| It will take a lot of time to get a diabetes test | 129 | 17.6 | 126 | 18.1 | 0.872 | The amount of time I would need to get a diabetes test is clearly specified | 607 | 76.6 | 435 | 73.7 | 0.406 |
| Even if I find out that I have diabetes I don't think I can afford the treatment | 132 | 17.0 | 101 | 15.5 | 0.595 | I am clearly told where I can access free diabetes testing | 558 | 71.4 | 407 | 65.0 | 0.084 |
| The pain from a diabetes test makes me reluctant to get tested for diabetes | 90 | 11.7 | 71 | 13.6 | 0.482 | The health screening tests can be held over weekends | 525 | 69.8 | 374 | 59.3 | **0.005** |
| I do not like to fast overnight which is needed for a proper health screening | 77 | 10.4 | 72 | 14.5 | 0.113 | My family and friends accompanied me for the health screening | 451 | 57.0 | 311 | 49.1 | **0.042** |
| I do not want to know if I have diabetes | 68 | 7.1 | 83 | 14.0 | **0.002** | I am given incentives in the form of supermarket vouchers | 430 | 52.3 | 307 | 50.0 | 0.555 |
| Knowing that I am at high risk for developing diabetes makes it difficult for me | 48 | 5.7 | 38 | 6.2 | 0.816 | The screening was held in association with workout sessions such as yoga classes | 415 | 52.1 | 299 | 49.5 | 0.515 |
|  |  |  |  |  |  | I am given incentives in the form of free gym classes | 440 | 51.1 | 284 | 46.1 | 0.206 |
|  |  |  |  |  |  | The screening was held in association with workshops on healthy cooking | 341 | 39.2 | 290 | 45.2 | 0.121 |

*p value less than 0.05 is considered significant (Chi-Square Test)
